# Supplementary material for: Multinuclear 1D and 2D NMR with 19F-Photo-CIDNP hyperpolarization in a microfluidic chip with untuned microcoil
Source: Nat Commun. 2023 Jun 30;14:3885. doi: 10.1038/s41467-023-39537-8 (PMC10313780; doi:10.1038/s41467-023-39537-8)
Supplement: Supplementary file 1 — Supplementary Information [file 41467_2023_39537_MOESM1_ESM.pdf]

SUPPLEMENTARY INFORMATION

Multinuclear 1D and 2D NMR with ^{19}F-Photo-CIDNP hyperpolarization in a microfluidic chip with untuned microcoil

M. Victoria Gomez,[1,]* Sander Baas,[2] Aldrik H. Velders.[1,2]*
1. IRICA, Department of Inorganic, Organic and Biochemistry, Faculty of Chemical Sciences and Technologies, Universidad de Castilla-La Mancha (UCLM), 13071 Ciudad Real, Spain
2. Laboratory of BioNanoTechnology, Wageningen University, 6700 EK Wageningen, The Netherlands
Send correspondence to mariavictoria.gomez@uclm.es or to aldrik.velders@wur.nl

Table of Contents:

Supplementary Figure 1. Detailed view of front & back of the microfluidic chip in a 3D printed-holder.(S-2)

Supplementary Table 1. Experimental data for the study of the photo-CIDNP efficiency as a function of p-fluorophenol / FMN (S-3).

Supplementary Table 2. Acquisition parameters for the multinuclear multidimensional NMR experiments (S-4).

Supplementary Figure 2. Photographs of the microfluidic chip integrated in the 3D printed holder (S-5).

Supplementary Figure 3. Calibration of the ^{19}F 90º pulse width in a hardware configuration for the excitation of up to three different nuclides (S-6).

Supplementary Figure 4. ^{19}F-^{13}C HMQC on neat TFE, ^{1}H decoupled mode, on the untuned spiral planar microcoil from Figure 6, main text (S-7).

Supplementary Figure 5. ^{19}F^{13}C-HMQC on neat TFE from the multinuclear multidimensional NMR experiments on the untuned spiral planar microcoil shown in Figure 6, main text (S-8).

Supplementary Figure 6. ^{19}F-^{13}C HMBC on neat TFE with ^{1}H decoupled mode on the untuned spiral planar microcoil shown in Figure 6, main text (S-9).

Supplementary Figure 7. ^{19}F-^{13}C HMBC on neat TFE from the multinuclear multidimensional NMR experiments on the untuned spiral planar microcoil shown in Figure 6, main text (S-10).

Supplementary Figure 8. Comparison of required NMR experimental time for a certain SNR between a photo-CIDNP-assisted untuned planar coil (this work) and an untuned planar coil (previous reports by us) (S-11).

Supplementary Figure 9. Photo-CIDNP ^{19}F NMR for 1 mM of p-fluorophenol. (S-12)

Supplementary Figures 10-14. Photo-CIDNP ^{19}F NMR for the calculation of the perceived concentration and signal enhancement (data from Figure 8) (S-13-17).

Supplementary Figure 15. Photo-CIDNP ^{19}F NMR of 0.5 mM of p-fluorophenol (S – 18)

Supplementary Figure 16. MRI images of the microfluidic chip channels (S – 19)

Supplementary Figure 17. ^{1}H^{19}F-HSQC on neat TFE (S – 20)

References (S-21).

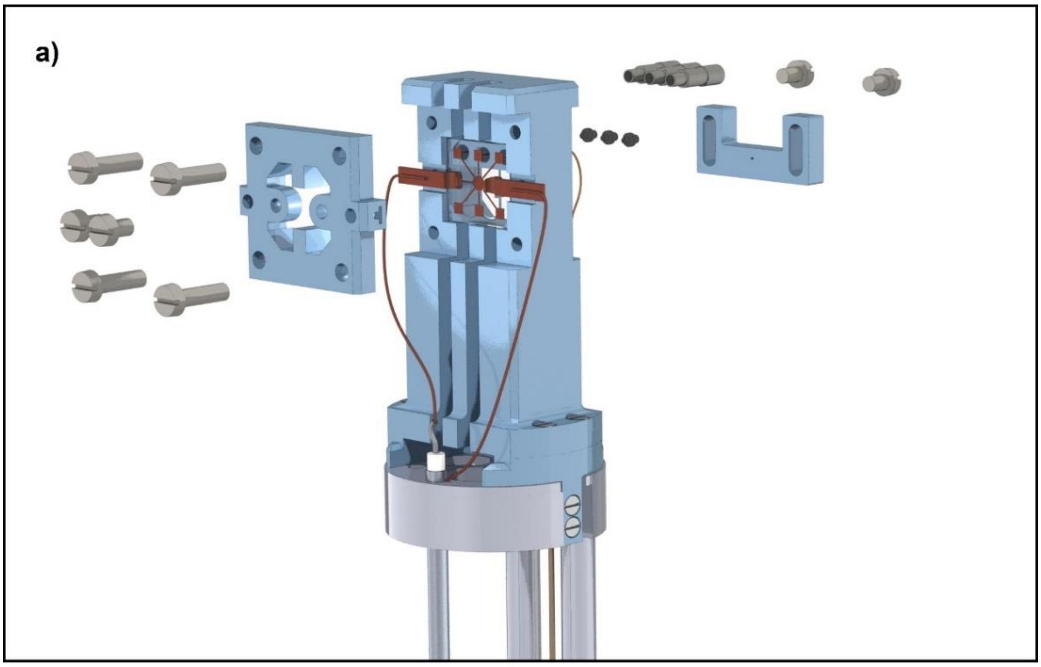

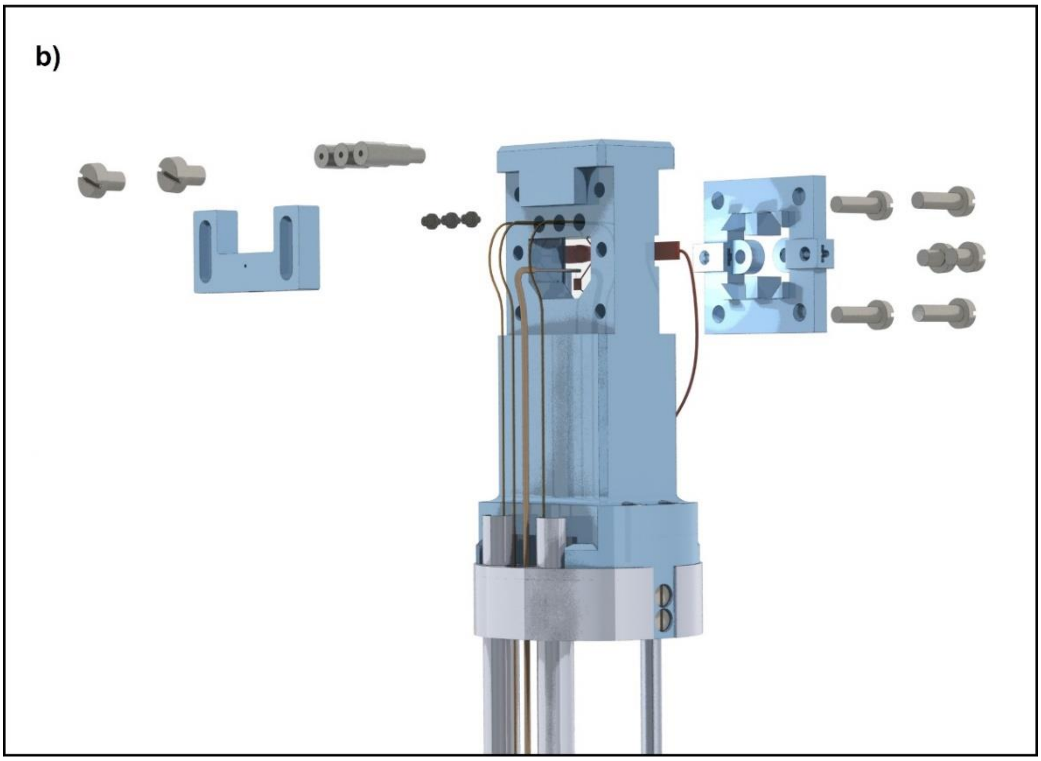

Supplementary Figure 1. Detailed view of the front and back side of the microfluidic chip integrated in the 3D printed-holder. **A**) Top Clamp, with screws for mounting the Top Clamp and for pressing the copper contact strips onto the NMR chip. The contact strips are still in place on the main holder. On the backside the three microfluidic ferrules and fiber slider holder can be seen. **B**) Backside of the same exploded view, where the three capillaries can be seen entering the threaded ferrule holes, as well as the optical fiber pointed towards the chip.

Supplementary Table 1. Experimental data for the study of the photo-CIDNP efficiency as a function of p-fluorophenol / FMN. Experimental data obtained for Figure 4d. Syringe in pump 1 contained 10 mM of *p*-fluorophenol and 300 mM of TFE. Syringe in pump 2 contained 10 mM of *p*-fluorophenol, 300 mM of TFE, 600 mM of TFP and 5 mM of FMN.

Pump 1 Flow rate	Pump 2 Flow rate	[TFP]	[FMN]	[p-fluorophenol] / [FMN]	Integral fluorophenol
2,0	0	--	--	--	--
1,80	0,2	62	0,43	23,2	67
1,80	0,2	61	0,42	23,6	71
1,75	0,25	78	0,54	18,5	82
1,70	0,3	81	0,56	17,7	83
1,70	0,3	87	0,60	16,5	87
1,70	0,3	96	0,66	15,0	93
1,65	0,35	111	0,77	12,9	96
1,65	0,35	116	0,80	12,4	81
1,65	0,35	137	0,95	10,5	85
1,60	0,4	120	0,83	23	82
1,50	0,5	140	0,97	10,3	73
1,40	0,6	191	1,33	7,5	73
1,30	0,7	209	1,45	6,9	81
1,20	0,8	250	1,74	5,8	64
1,1	0,9	323	2,24	4,5	70
1	1	346	2,40	4,2	76
0,75	1,25	405	2,81	3,5	67
0,5	1,5	485	3,37	3,0	52
0,25	1,75	528	3,67	2,7	57
0	2	655	4,55	2,2	58
0	2	720	5	2,0	71
0	2	735	5,1	2,0	66

Supplementary Table 2. Acquisition parameters for the multinuclear multidimensional NMR experiments: Acquisition parameters for the NMR experiments shown in Figures 5, 6 and 7. Sw and sw1 are the spectral windows in the direct and indirect detection, respectively.

Sample	Experiment	Number of increments in indirect detection	Number of scans per increment	sw	sw1
Trifluoroethanol & Trifluoropropanol (Figure 5 right)	$^{19}F^{1}H$-HMQC	128	4	56298	6999
Trifluoroethanol (Figure 6 A)	$\{^{1}H\}^{19}F^{13}C$-HMBC	256	64	56298	31446
Trifluoroethanol (Figure 6 B)	$^{19}F^{13}C$-HMBC	256	64	56298	31446
Trifluoroethanol (Figure 6 C)	$\{^{1}H\}^{19}F^{13}C$-HMQC	64	128	14000	11999
Trifluoroethanol (Figure 6 D)	$^{19}F^{13}C$-HMQC	64	128	14000	11999
13 mM p-fluorophenol, 1.1 mM FMN, 300mM TFE (Figure 7 D)	2D Photo-CIDNP ^{19}F-^{1}H HMQC	16	32	49200	16000
1.0 mM p-fluorophenol, 0.024 M TFE (Figure 7 E)	^{19}F-^{1}H HMQC	20	32	113636	10003

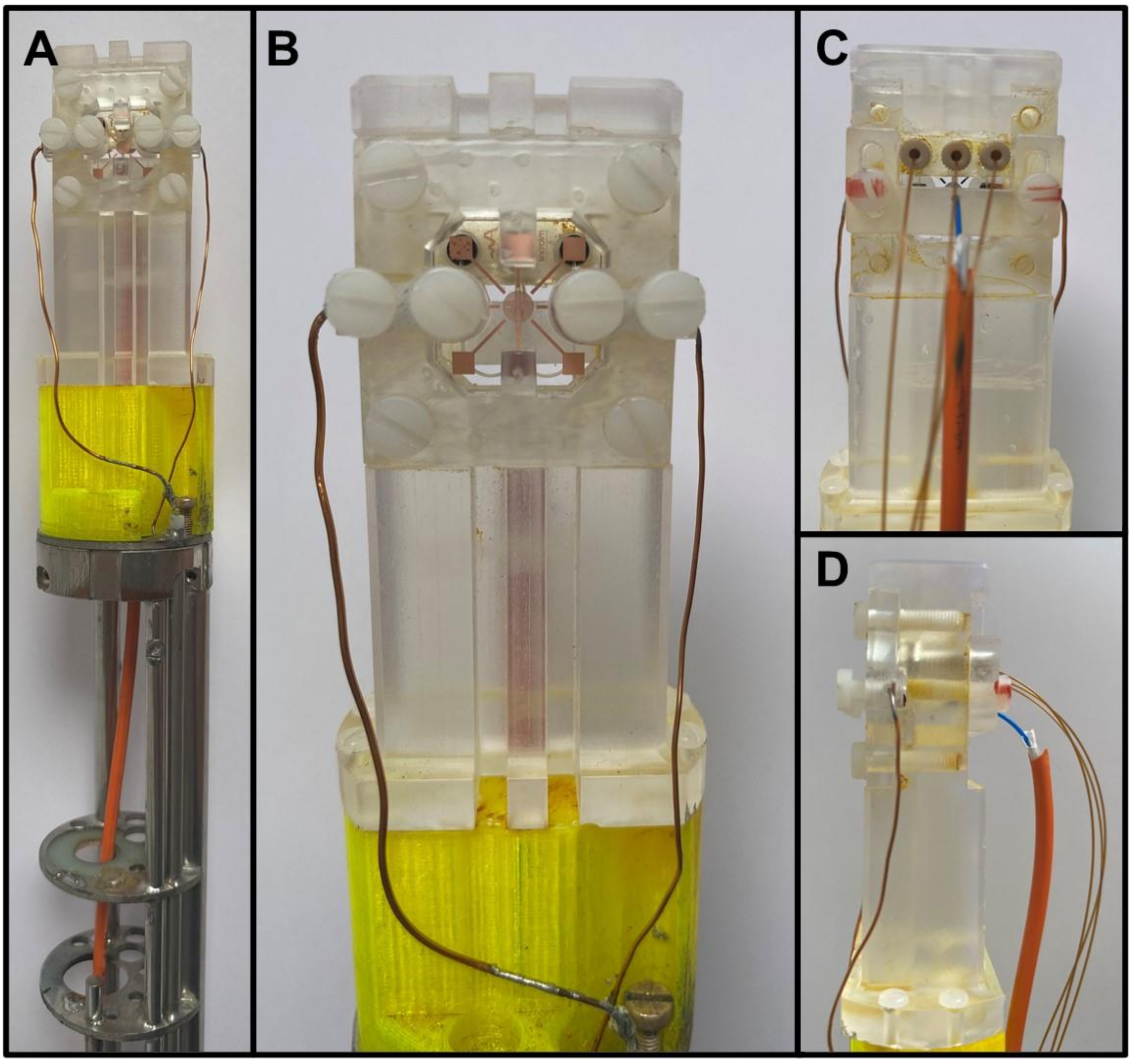

Supplementary Figure 2. Photographs of the microfluidic chip integrated in the 3D printed-holder. A) Front view that visualizes the decommissioned, non-functional probe that was used as a probe platform. **B**) Zoom of the front view of the chip-holder showing the planar spiral coil integrated on top of the microfluidic chip and the electronic connections. **C)** Back side of the chip-holder showing the fused silica capillaries for the microfluidic connections and the optical fiber to illuminate the NMR active volume. **D)** Cross-section view of the chip-holder showing one electronic connection, the fused silica capillaries for the microfluidic connections and the optical fiber to illuminate the NMR active volume.

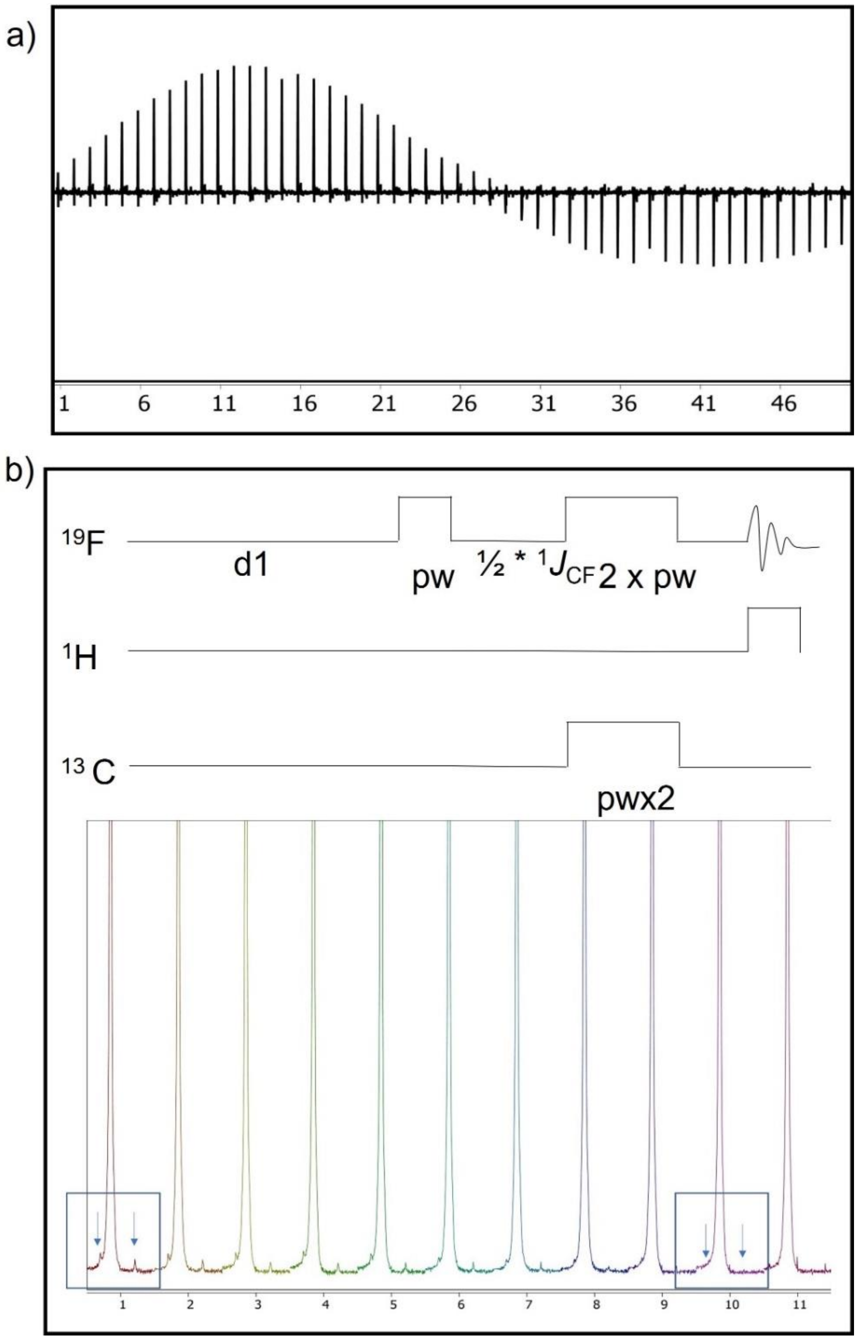

Supplementary Figure 3. Calibration of the ^{19}F 90° pulse width on neat TFE in a hardware configuration with two combiners (Figure 2e, main text) that allows the excitation of up to three different nuclides. (a): 90° pulse width calibration for ^{19}F with an experiment acquired in the arrayed mode (50 steps, starting from 0, increments of 1 µs). The first null of the ^{19}F signal corresponds to the 180° pulse width for ^{19}F (pw_{180}=29 µs). The acquisition parameters are the following: preacquisition delay= 3 s, acquisition time= 0.5 s and number of scans= 2 s. **(b):** 90° pulse width calibration for ^{13}C with an experiment acquired in the arrayed mode (11 steps, starting from 0, increments of 15 µs). The first null for the ^{13}C satellite peaks of the TFE ^{19}F signal (step=10) corresponds to the 90° pulse width for ^{13}C (pwx= 135 µs). The experiment was run with ^{1}H decoupling. The acquisition parameters are the following: $^{1}J_{CF}$ = 290 Hz, decoupling power= 0.2 W, ^{19}F pulse width= 14.5 µs, preacquisition delay= 3 s, acquisition time= 0.5 s and number of scans= 64.

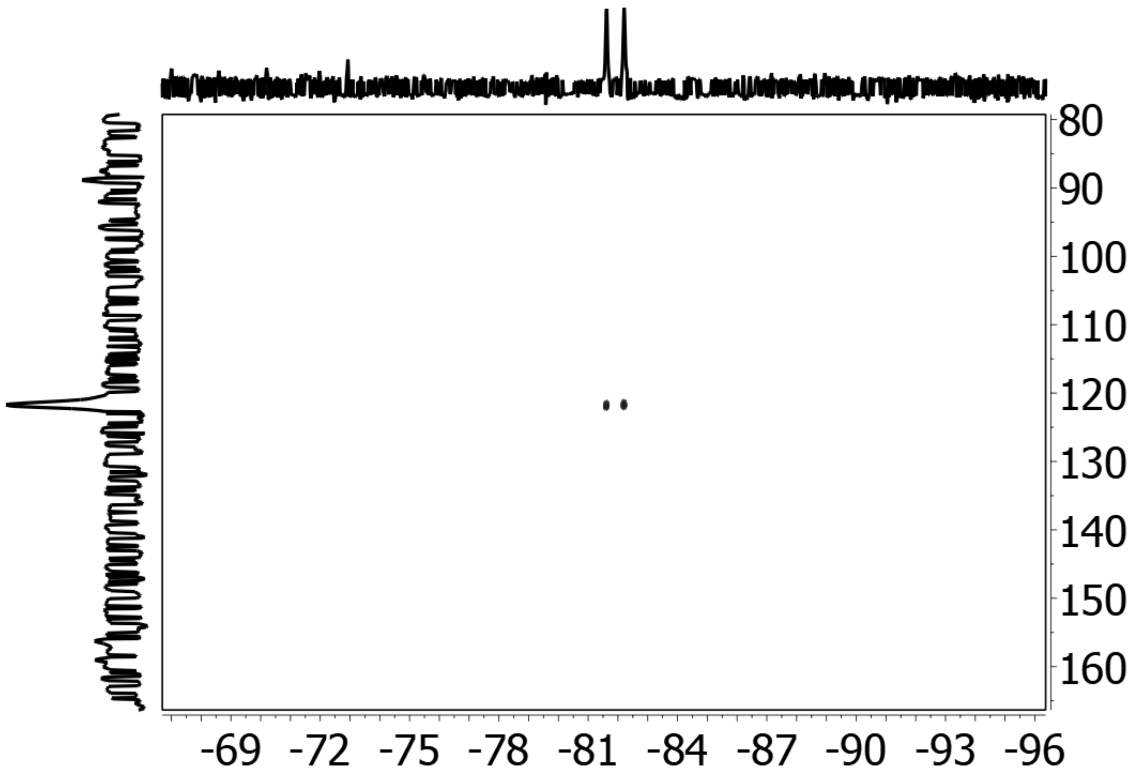

Supplementary Figure 4. ^{19}F-^{13}C HMQC on neat TFE from the multinuclear multidimensional NMR experiments on the untuned spiral planar microcoil shown in Figure 6, main text.

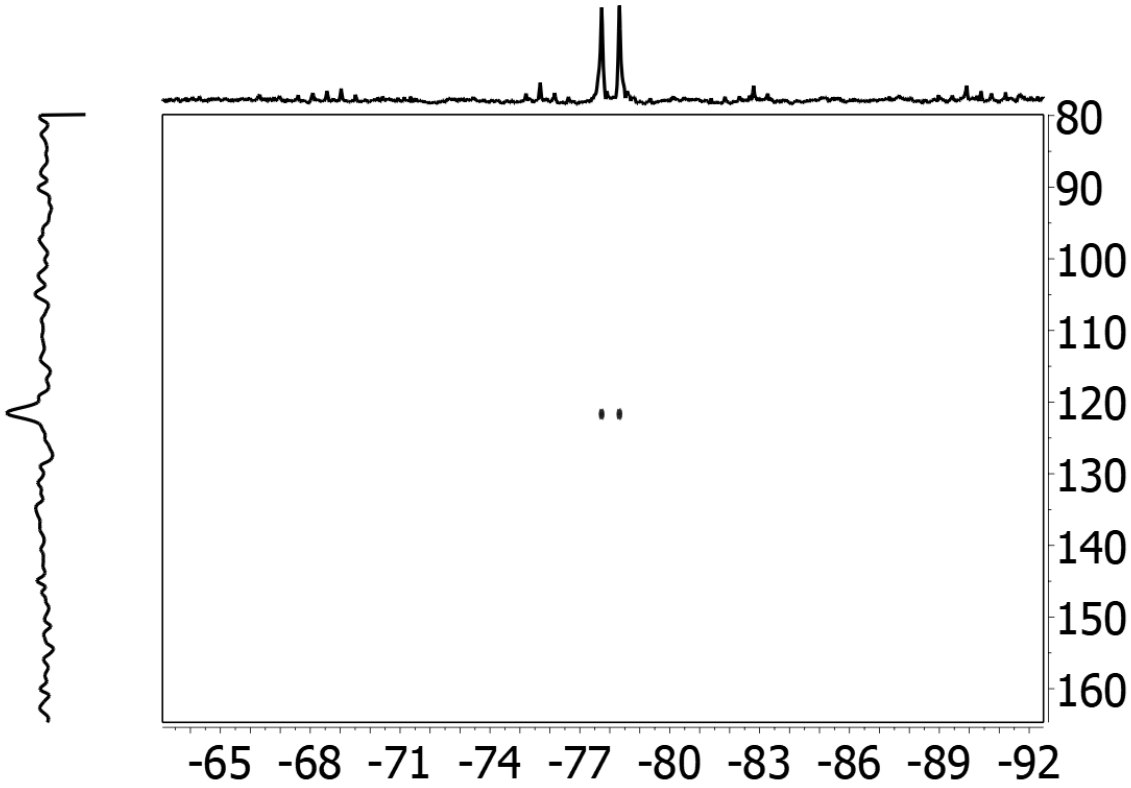

Supplementary Figure 5. ^{19}F-^{13}C HMQC on neat TFE with ^{1}H decoupled mode from the multinuclear multidimensional NMR experiments on the untuned spiral planar microcoil shown in Figure 6 main text.

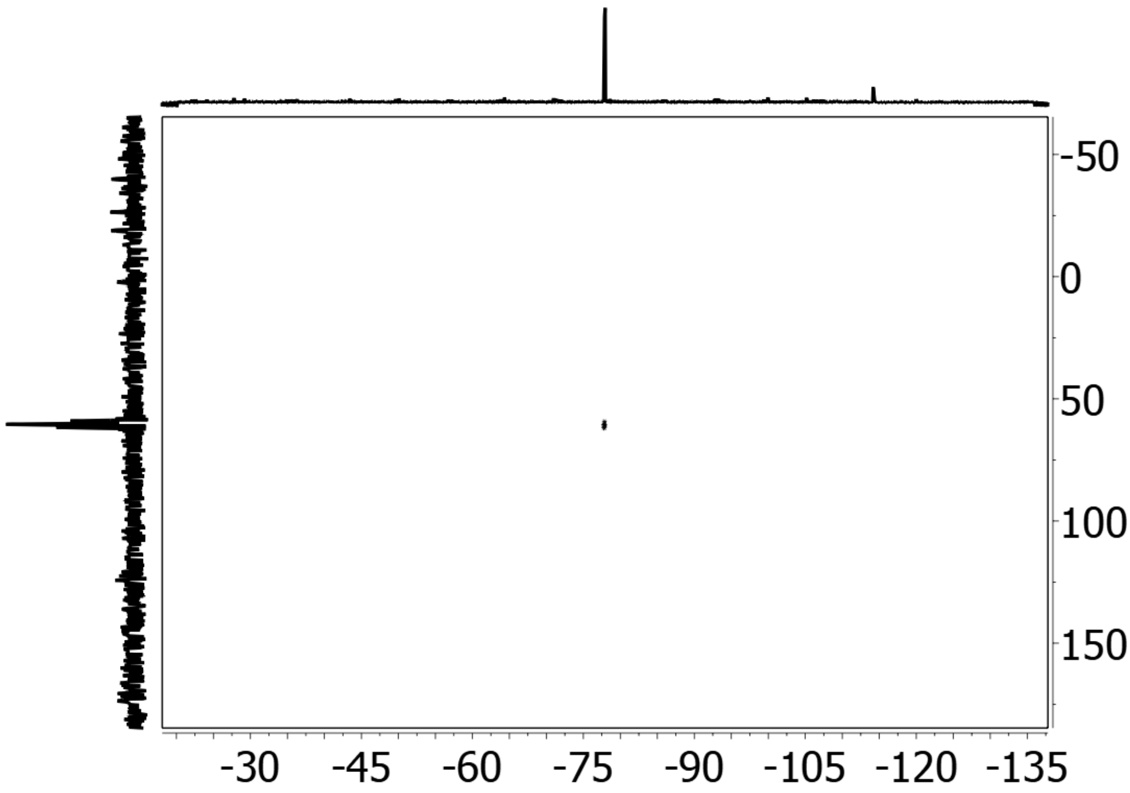

Supplementary Figure 6. $^{19}F^{13}C$-HMBC on neat TFE from the multinuclear multidimensional NMR experiments on the untuned spiral planar microcoil shown in Figure 6, main text.

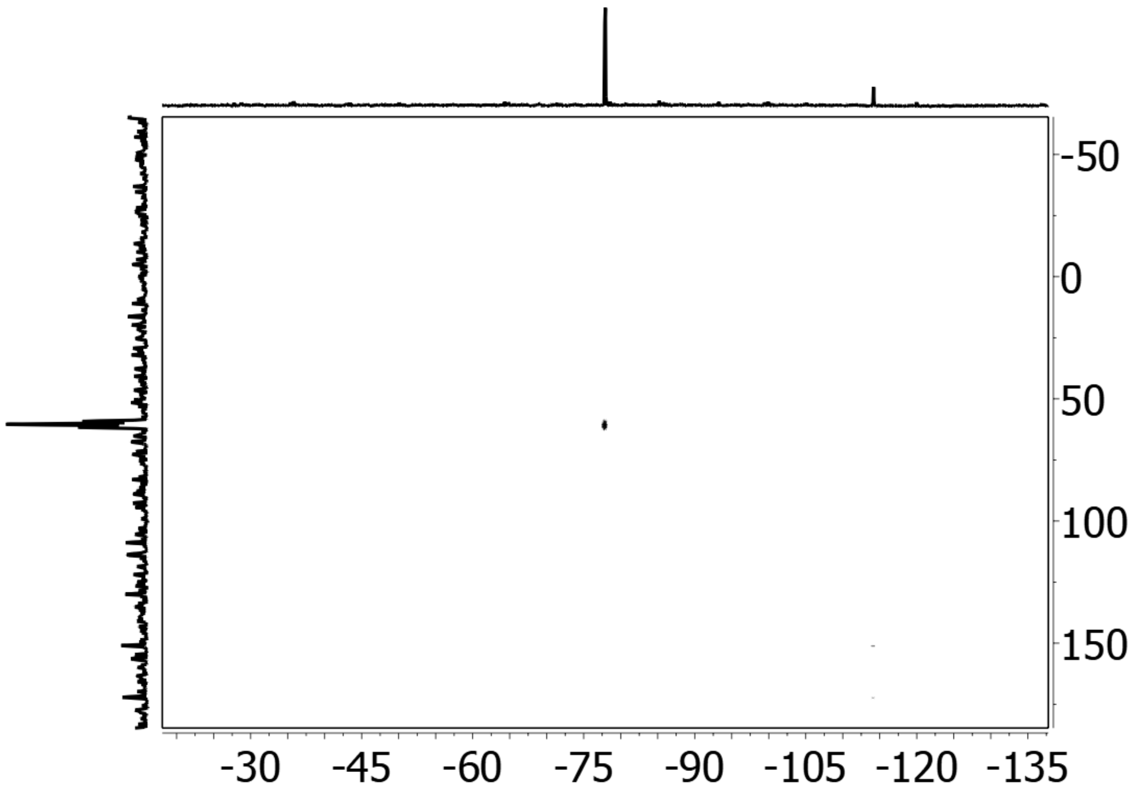

Supplementary Figure 7. ^{19}F-^{13}C HMBC on neat TFE with ^{1}H decoupled mode from the multinuclear multidimensional NMR experiments on the untuned spiral planar microcoil from Figure 6, main text.

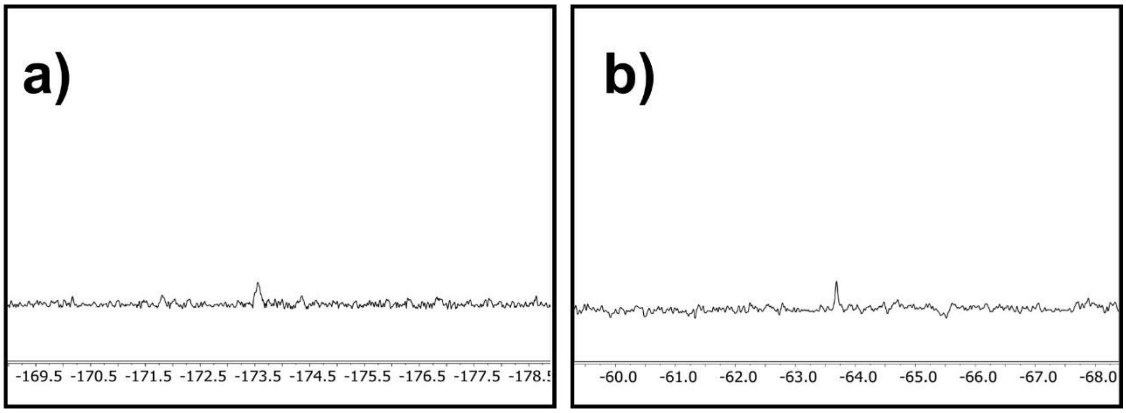

Supplementary Figure 8. Comparison of required NMR experimental time for a certain SNR between a photo-CIDNP-assisted untuned **planar coil (this work) and an** untuned **planar coil from our previous work.[1] A):** ^{19}F NMR photo-CIDNP spectrum of 80 picomole of 5-fluorouracile (3.2 mM of 5-fluorouracile and 0.2 mM of FMN) in stopped flow (SNR= 5.3 measured with the MestRenova script). The number of scans is 100 and the NMR experiment time is 1 min 41 seconds. Line broadening is 10. **B)**: ^{19}F-NMR spectra of 25 picomole of trifluoromethylphenol from our previous work (SNR = 6,0 measured with the MestRenova script).[1] The number of scans is 16000 and the NMR experiment time is 6h 43 min. Line broadening is 10.

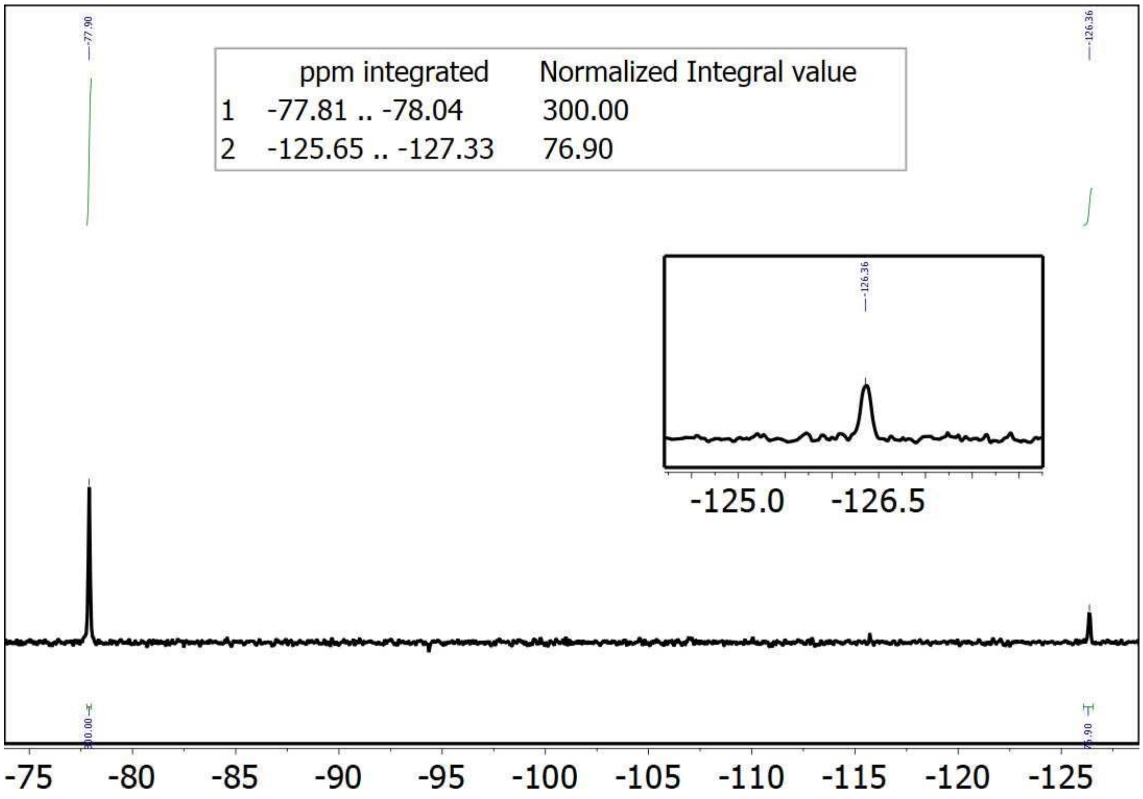

Supplementary Figure 9. Photo-CIDNP ^{19}F NMR for 1 mM of p-fluorophenol. NMR spectrum of 1mM of p-fluorophenol and 0.1 mM of FMN in the presence of 300 mM of TFE. The integral of TFE has been normalized to 300, resulting in an integral value for the *p*-fluorophenol of 76.90, which corresponds to a signal enhancement of 230 times and a perceived concentration of p-fluorophenol of 230 mM. The number of scans is 64.

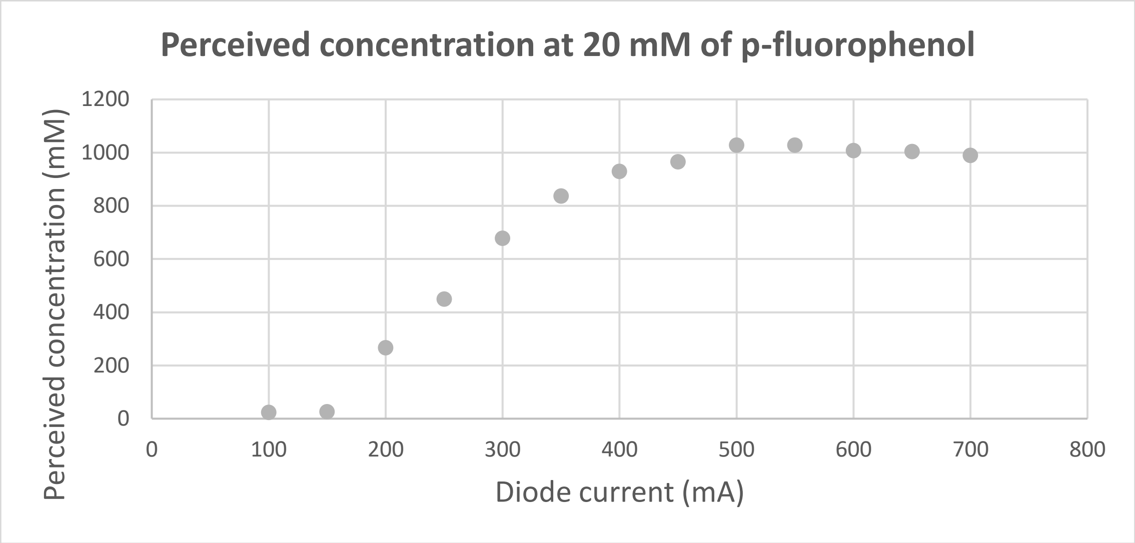

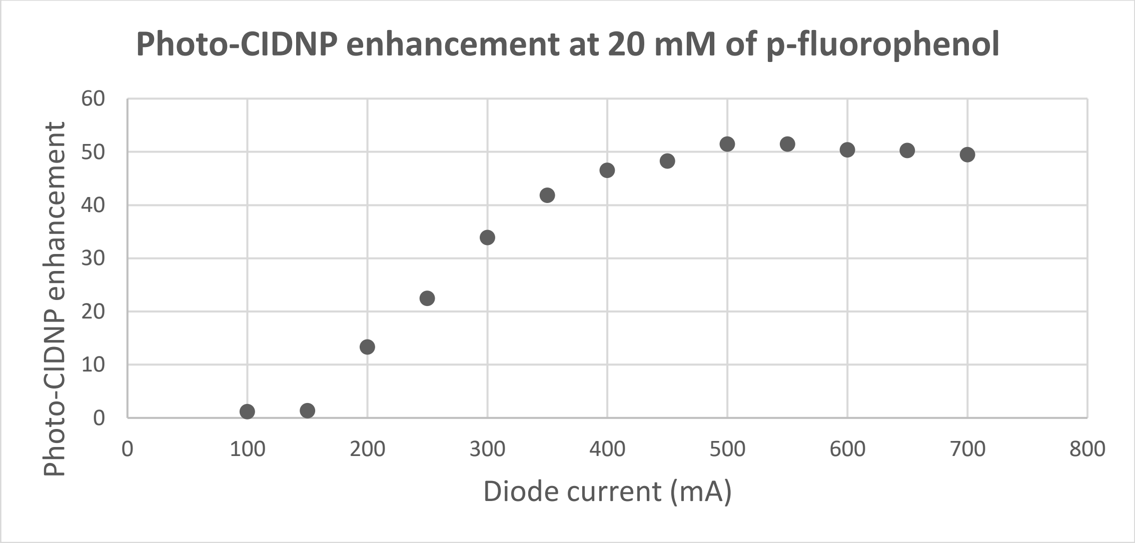

Supplementary Figure 10— Perceived concentration (top) and photo-CIDNP enhancement (bottom) for 20 mM of p-fluorophenol in the presence of 2 mM of FMN and 300 mM of TFE as internal standard, as a function of the laser diode current (mA).

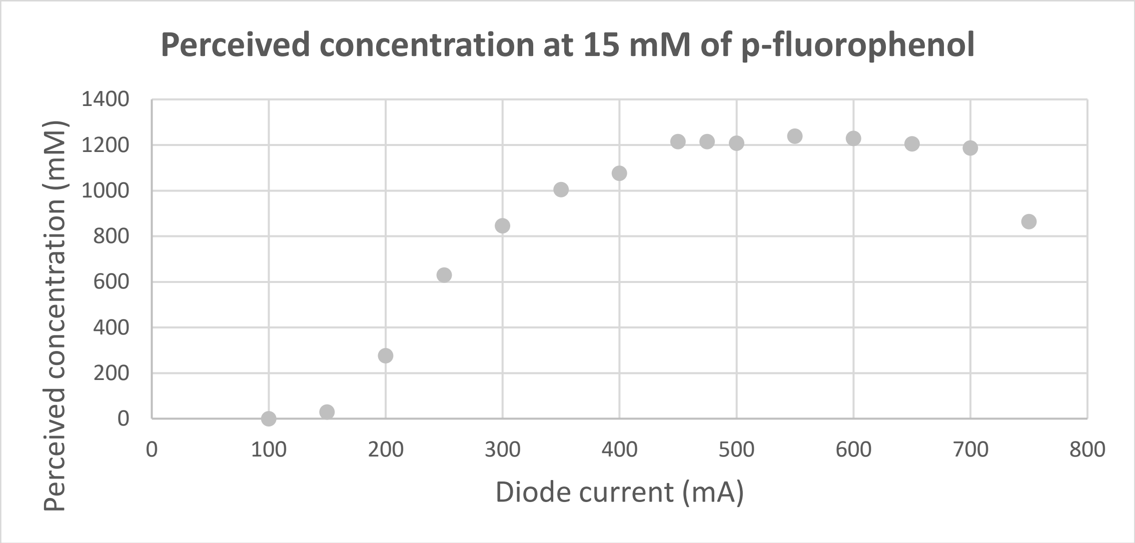

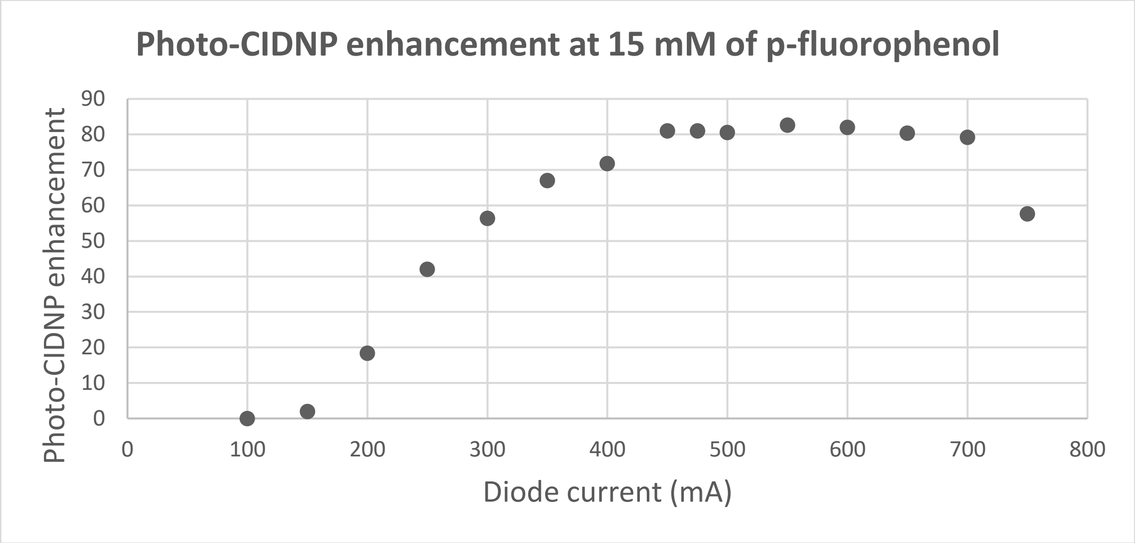

Supplementary Figure 11—Perceived concentration (top) and photo-CIDNP enhancement (bottom) for 15 mM of p-fluorophenol in the presence of 1.5 mM of FMN and 300 mM of TFE as internal standard, as a function of the laser diode current (mA).

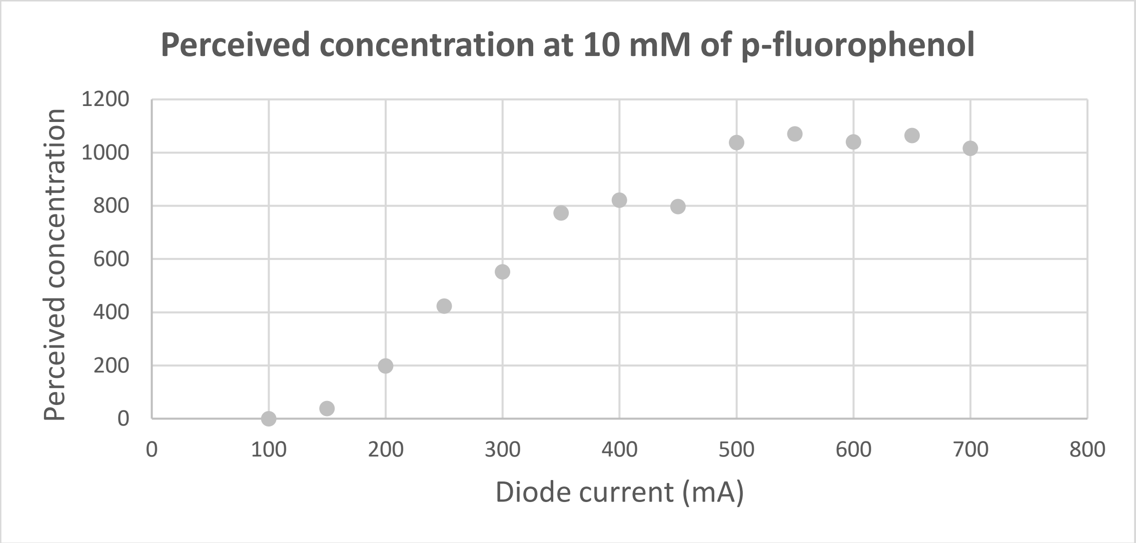

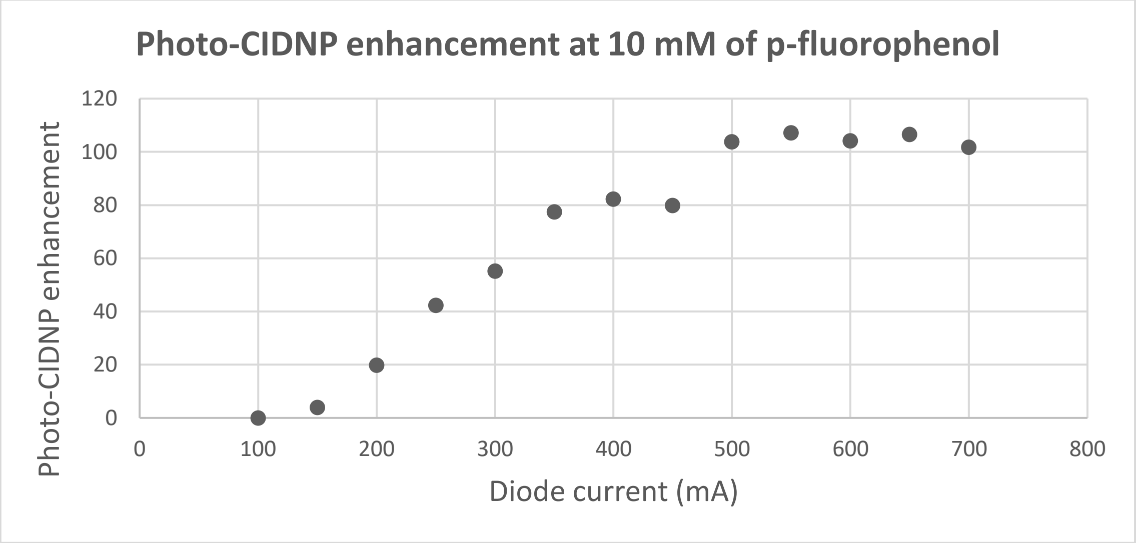

Supplementary Figure 12—Perceived concentration (top) and photo-CIDNP enhancement (bottom) for 10 mM of p-fluorophenol in the presence of 1.0 mM of FMN and 300 mM of TFE as internal standard, as a function of the laser diode current (mA).

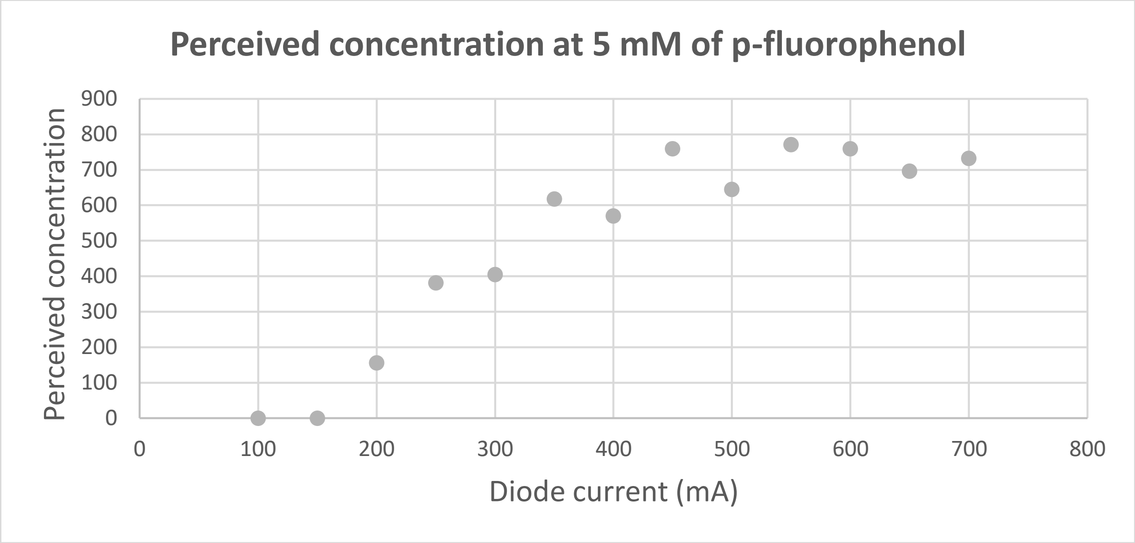

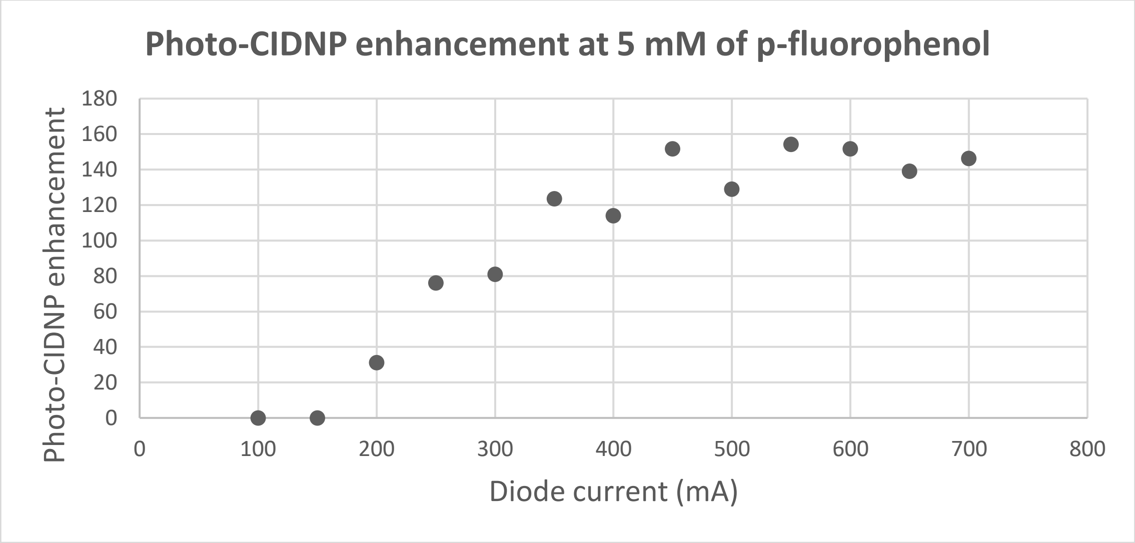

Supplementary Figure 13—Perceived concentration (top) and photo-CIDNP enhancement (bottom) for 5 mM of p-fluorophenol in the presence of 0.5 mM of FMN and 300 mM of TFE as internal standard, as a function of the laser diode current (mA).

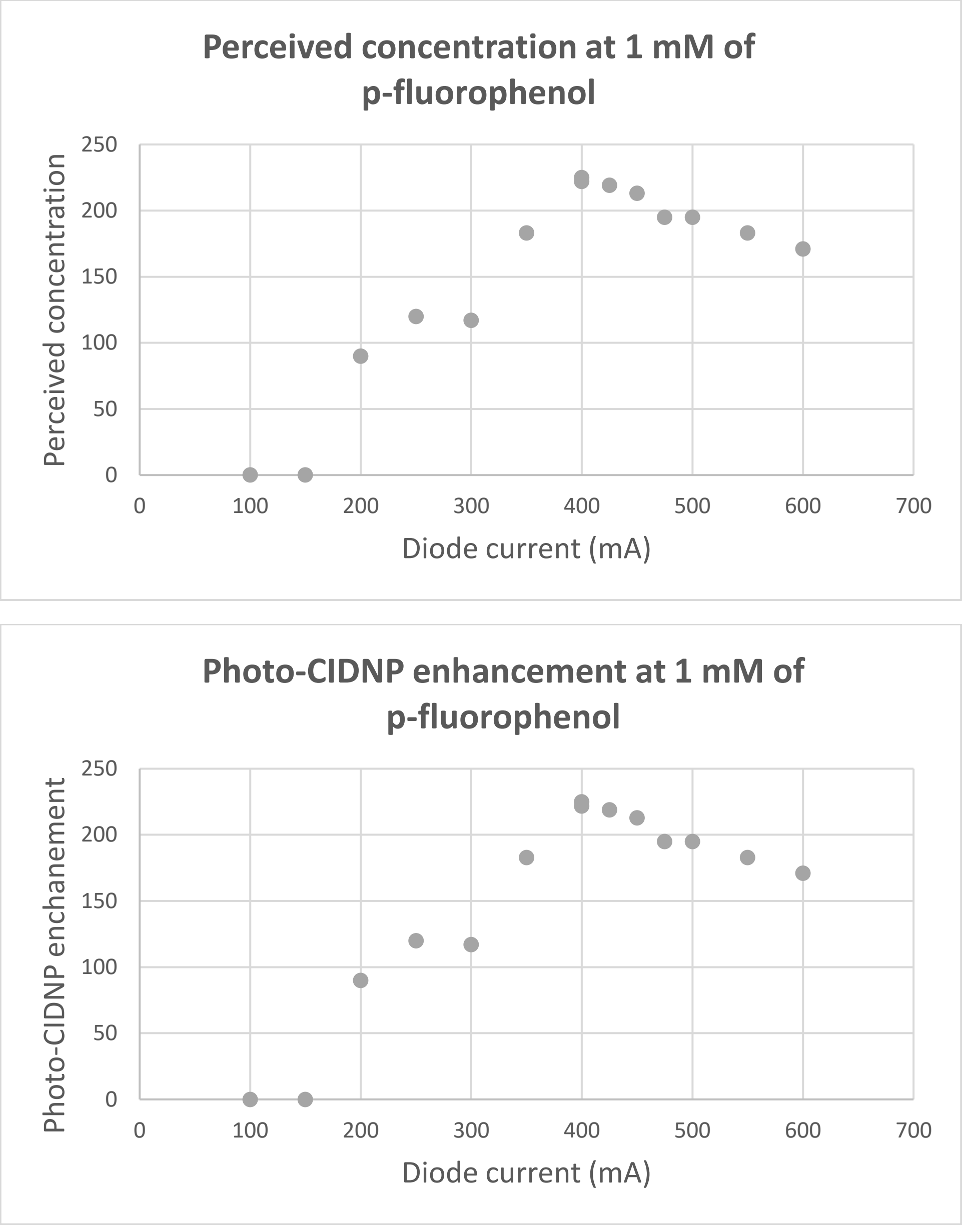

Supplementary Figure 14—Perceived concentration (top) and photo-CIDNP enhancement (bottom) for 1 mM of p-fluorophenol in the presence of 0.1 mM of FMN and 300 mM of TFE as internal standard, as a function of the laser diode current (mA).

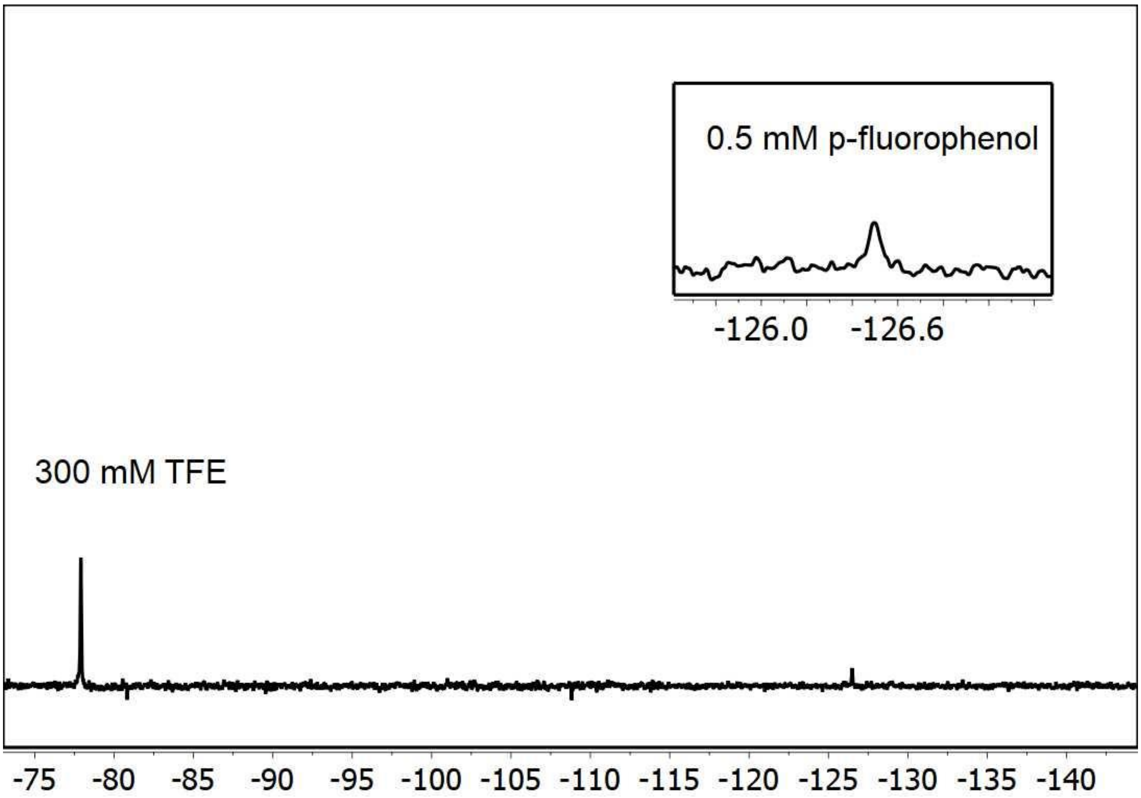

Supplementary Figure 15. Photo-CIDNP ^{19}F NMR of 0.5 mM of p-fluorophenol. NMR spectrum of 0.5 mM of p-fluorophenol and 0.005 mM of FMN in the presence of 300 mM of TFE.

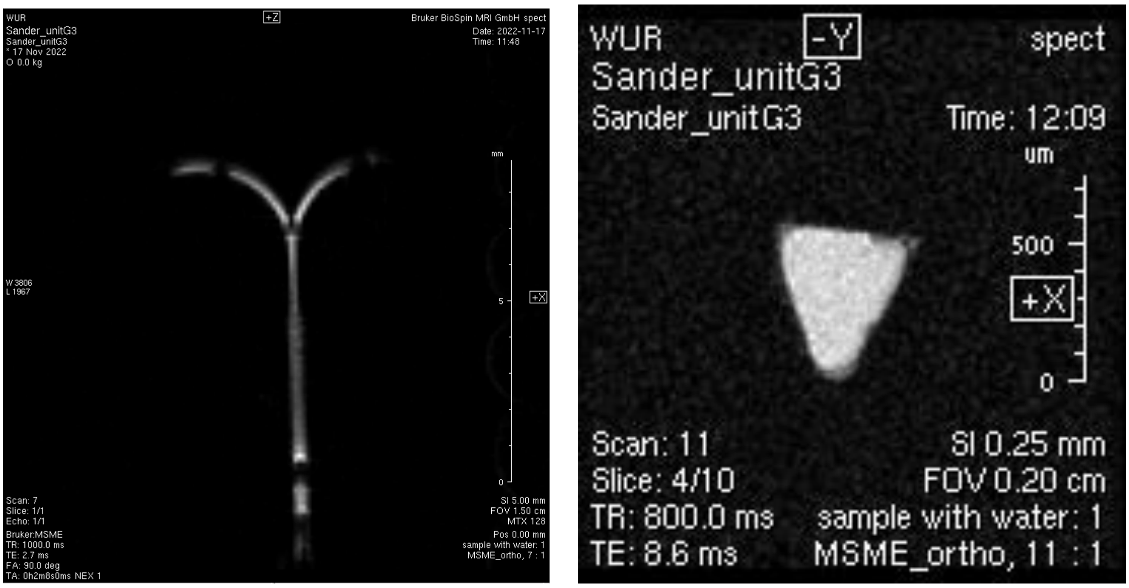

Supplementary Figure 16. Micro-MRI orthogonal slices of a MSME experiments on a sacrificed microfluidic NMR chip filled with water, revealing the geometry and dimensions of the channels.

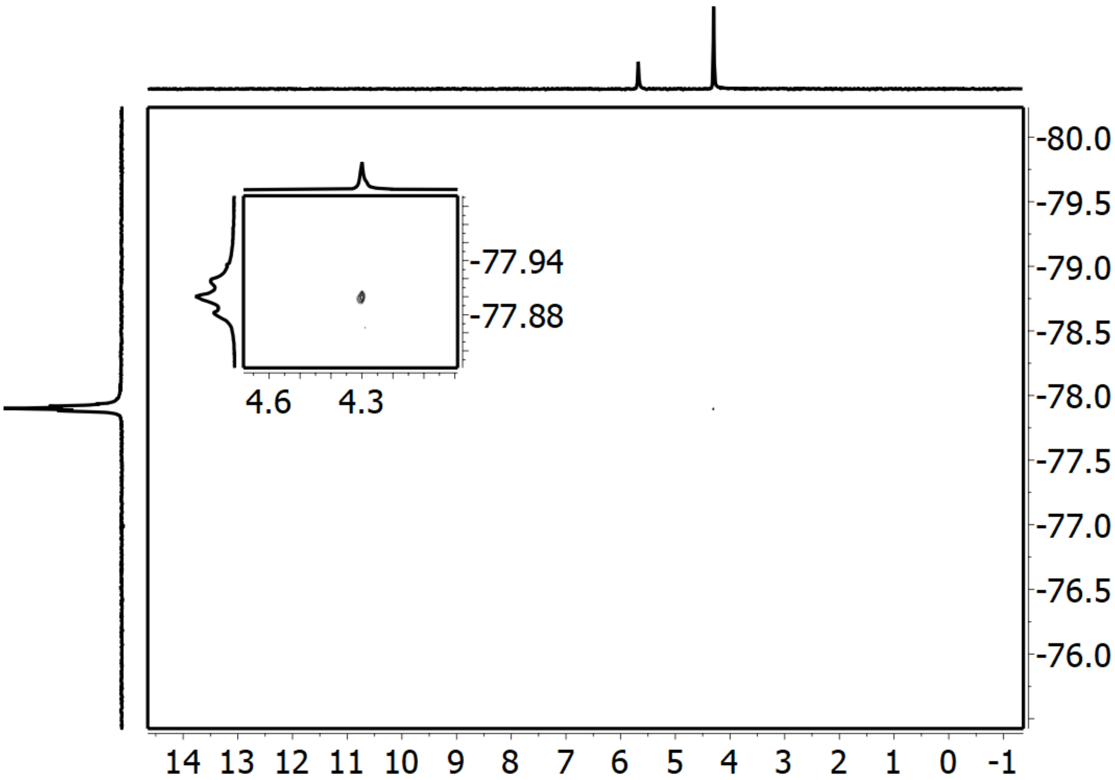

Supplementary Figure 17. 2D $^{1}H^{19}F$-HSQC NMR on neat TFE in stopped flow. The total experiment time is 80 minutes. The acquisition parameters are 4 as number of scans and 512 as number of increments.

REFERENCES

1. Fratila, R. M., Victoria Gomez, M., Sykora, S. & Velders, A. H. Multinuclear nanoliter one-dimensional and two-dimensional NMR spectroscopy with a single non-resonant microcoil. *Nat. Commun.* **5**, 3025 (2014).
